# DIM: A diffusion instability measure for MRI quality assurance

**DOI:** 10.1002/mrm.70012

**Published:** 2025-08-10

**Authors:** Tim Schmidt, Zoltan Nagy

**Affiliations:** ^1^ Laboratory for Social and Neural Systems Research (SNS Lab) University of Zurich Zurich Switzerland; ^2^ Institute for Biomedical Engineering ETH Zurich and University of Zurich Zurich Switzerland

**Keywords:** diffusion, instability, magnetic field monitoring, MRI, quality assurance

## Abstract

**Purpose:**

The aim of the presented work is to develop a diffusion instability measure (DIM) that can be used in quality assurance.

**Methods:**

Single‐shot, spin‐echo, echo‐planar imaging HARDI diffusion data sets were collected on a spherical silicone oil phantom with 64 different diffusion directions on a 3T Philips Achieva and a 3T Siemens Cima.X scanner with similar acquisition protocols. A few data sets on the Philips Achieva included concurrent magnetic field monitoring. A correlation coefficient matrix among the diffusion directions for each data set was calculated, and subsequently, one minus its eigenratio was defined as the DIM.

**Results:**

The DIM values ranged between about 5000 and 280 000 ppm across the data sets. The worst and best image quality—and hence the highest and lowest DIM values—were observed for b‐value = 4000 s/mm^2^ on the Philips Achieva and for b‐value = 500 s/mm^2^ on the Siemens Cima.X without concurrent field monitoring, respectively.

**Conclusion:**

DIM is a sensitive tool for evaluating image quality in HARDI scans on a quantitative basis. It is simple to implement without the need for hardware or software modifications.

## 1 INTRODUCTION

The importance of reproducible research results has been gaining deserved attention both generally in science and more specifically in MRI.^1^, ^2^ Achieving such a goal requires a spectrum of effort, including the regular quality assurance (QA) of the equipment that produces the data. Various QA protocols exist in MRI,^3^ but few are developed specifically for diffusion MRI (dMRI) despite it being one of the most important workhorses for studying the neuroanatomy of the human brain.^4^ Although it has opened new ways to scrutinize neuronal connectivity by mapping white‐matter tracts,^5^, ^6^ and delivered new diagnostic tools for detecting and investigating neuronal diseases,^7^, ^8^ dMRI data are inherently sensitive to various unwanted sources of error (e.g., eddy currents that are generated by the diffusion‐encoding gradients and can adversely affect the imaging gradients).^9^, ^10^, ^11^, ^12^

A previously proposed quality assurance protocol for dMRI data, RAPID, uses a dodecane‐filled phantom and an acquisition protocol with several b‐values from which to calculate various QA measures.^13^ Although the protocol is fairly comprehensive, it does not cover all aspects of dMRI data quality. For example, while the choice of dodecane for the phantom filling is appropriate because it mimics the diffusive properties of brain white matter well, very high b‐values cannot be used as the signal‐to‐noise ratio (SNR); hence, the reliability of the QA measures will progressively diminish. Furthermore, the protocol assumes that the images are free from artifacts (or artifacts can be mitigated) other than those of interest (i.e., the voxel‐wise signal intensity offset due to gradient nonlinearity and the geometric distortions due to eddy currents).

Here we put forth a complementary QA measure that utilizes a silicone oil phantom. Silicone oil offers practically no diffusion; hence, the individual diffusion‐weighted images (DWIs) are expected to have nearly identical voxel‐wise intensity as the b_o_ image. As such, the dMRI data set can be considered a “time series” (as in functional MRI [fMRI]) and used to assess the level of shot‐to‐shot variability (e.g., changing image ghosts, radiofrequency [RF] instability, parallel‐imaging reconstruction errors). Evaluation of the data relies on a recently introduced fMRI temporal instability measure (TIM). Because it is used for dMRI data, it is called a diffusion instability measure (DIM).

The present work demonstrates the utility of DIM in assessing data quality of various dMRI data sets from different MRI scanners, with and without magnetic field monitoring and up to b‐values of 10 000 s/mm^2^.

## 2 METHODS

### 2.1 The proposed DIM

**FIGURE 1:**
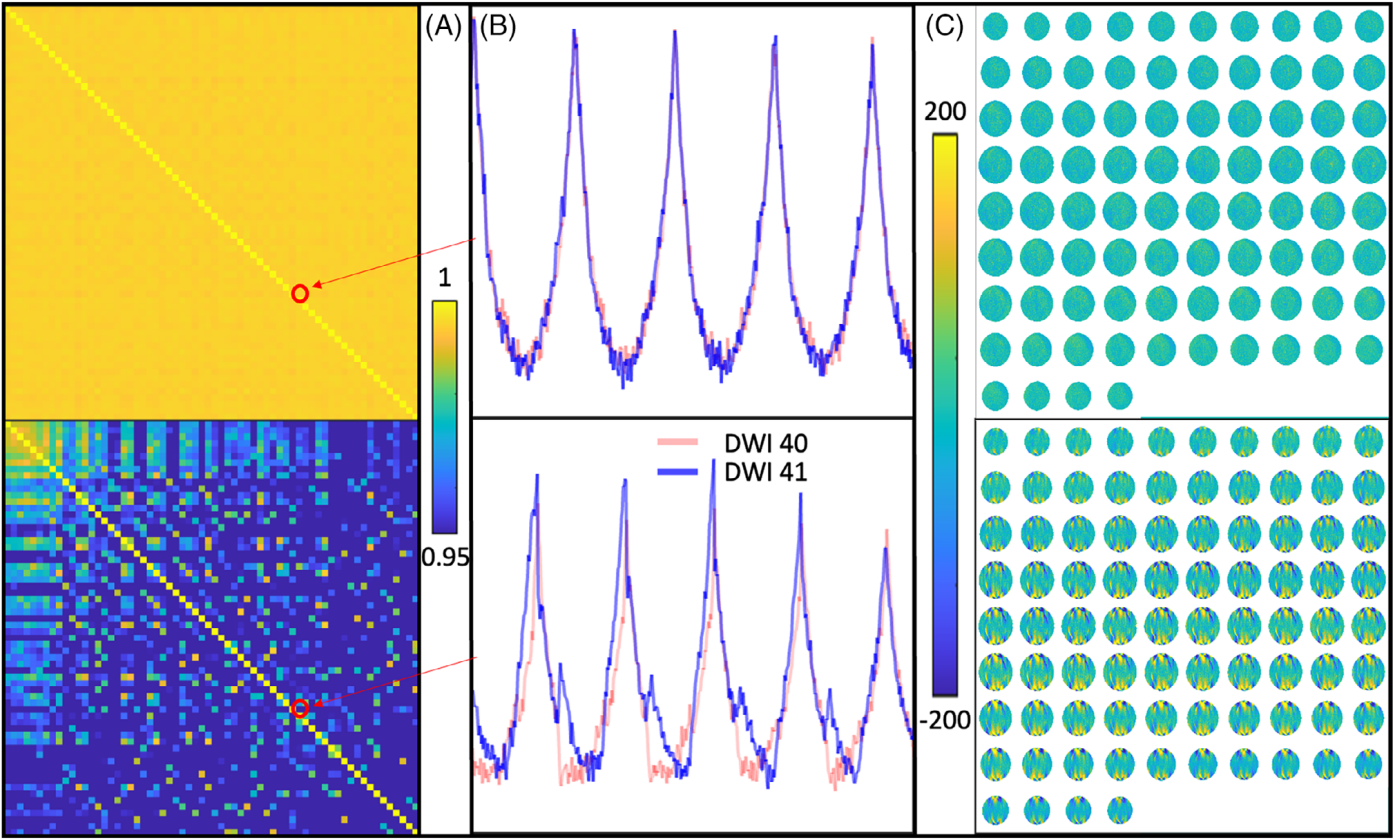
Illustration of diffusion instability measure (DIM) on representative examples. (A) Correlation coefficient matrices of a good (*top row*) and bad (*bottom row*) data set. The red circles highlight the correlation coefficient between the 40th and 41st DWI as computed from the corresponding full undulating curves (i.e., the reshaped signal voxel intensities in the columns of matrix $\hat{X}$), the short segments of which are illustrated in (B). (C) Tiled difference images between the 40th and 41st diffusion direction demonstrate the imaging data quality that DIM quantifies.

The details of calculating TIM on an fMRI time series are provided in Schmidt and Nagy.^14^ Next is a brief summary of applying this measure to a high angular resolution diffusion imaging (HARDI)^15^ data set collected on a silicone oil phantom. After extracting all the voxels from each diffusion‐weighted image (DWI), the data are reshaped into a two‐dimensional matrix, $X\in {\mathbb{R}}^{N_{v}\times N_{d}}$, with $N_{v}\approx 8*10^{5}$ voxels covering almost the entire silicone oil phantom and $N_{d}$ different diffusion‐encoding directions, the latter representing “time” (i.e., the successive DWIs; see Figure 1B for representative example segments of the undulating curves of the extracted voxel‐wise signal intensity $X_{:,40}$ and $X_{:,41}$), analogous to echo‐planar‐imaging volumes in an fMRI time series.

The related matrix, $\hat{X}$, contains the data after each column was centered with its own mean and normalized by its own standard deviation. Each entry, $c_{i,j}$ of the $N_{d}\times N_{d}$ matrix, $C={\hat{X}}^{t}\hat{X}$, contains the correlation coefficients between the *i*th and *j*th diffusion‐encoding directions (see Figure 1A). We define DIM as

(1) $$\mathrm{DIM}=1-\frac{\max_{i\in \left\{1,\ldots ,N_{d}\right\}}\lambda_{i}}{\sum_{i=1}^{N_{d}}\lambda_{i}}$$

where $\lambda_{i}$ are the eigenvalues of C. A useful interpretation is that if the first image were a good representation of the remaining $N_{d}-1$ DWIs (i.e., a data set without variability), there would be a single principal component, and the DIM value would be near zero (not zero because thermal noise always exists). Conversely, if the DWIs were maximally different from each other (i.e., the correlation coefficient of any two DWIs near zero), C would be close to the identity matrix and DIM would be close to 1. The value of DIM for a practically useful data set is close to zero (i.e., images look similar); hence, for practical usefulness, DIM is given in parts per million (ppm) and displayed across all acquired data in logarithmic scale for better visual comparison.

Figure 1A,B illustrates these considerations. The discrepancies between the red and blue curve in Figure 1B (bottom plot) already suggest that a different ghosting level might be a contributing factor for the lower correlation coefficient. This is further supported by the mosaic difference images between the 40th and 41st DWI in Figure 1C.

DIM inherently depends on the number of diffusion directions, $N_{d}$, and the number of voxels inside the volume of interest, $N_{v}$. The minimum $N_{d}$ required for a reliable DIM value cannot be determined a priori, because instability can in principle occur any time. Therefore, it is recommended that the length of the QA data acquisition be representative of the experiments usually performed. On the other hand, because DIM is calculated on a volume of interest that encompasses the entire phantom, the calculated value will be stable with respect to $N_{v}$, which is usually in the range of 10^5^ voxels. This we confirmed with simulations where we removed up to 20% of the voxels (1%, 2%, 3%, 5%, 10%, 20%) and computed the resulting mean $\mathrm{DI}M_{\mathrm{sim}}$ and its standard deviation across 100 resamples (Figure S1).

### 2.2 Data acquisition

We acquired several single‐shot, spin‐echo, EPI HARDI^15^, ^16^ data sets on two different 3T MRI scanners (Philips Achieva, Best, Netherlands; and Siemens Cima.X, Erlangen, Germany), using a spherical phantom with a diameter of 17 cm and filled with silicone oil (AK 500; Wacker Chemie AG, Munich, Germany).

On the Philips Achieva scanner, we used (i) the vendor's 32‐channel receive‐only head coil with and without f0 stabilization (f0S), where the purpose of f0S is to measure and follow resonance frequency shifts during image acquisition and reduce signal drifts, or (ii) a 16‐channel NeuroCam coil (Skope AG, Zurich, Switzerland) that enables concurrent monitoring of the magnetic field perturbations. Each scan included 64 diffusion directions sampled uniformly over a hemisphere starting at its apex, a b_0_ image with 1.4‐mm^3^ isotropic voxels, 84 slices, slice repetition time (TR) = 199 ms, and sensitivity encoding acceleration factor = 3.^17^ A total of 20 DWI data sets were collected, of which seven were performed twice (i.e., on manufacturer coil and NeuroCam with field monitoring) with b‐values between 500 to 10 000 s/mm^2^ and six runs with a constant b‐value of 1000 s/mm^2^ only with the vendor coil, but with the f0S stabilization option switched on and off alternately. The echo time (TE) with increasing b‐value ranged between 76 ms (b‐value 500 s/mm^2^) and 151 ms (b‐value 10 000 s/mm^2^). Moreover, the field‐monitored scans also included a low‐resolution (3 × 3 × 1.4 mm^3^), multiple gradient‐echo (five echoes; first TE = 4.6 ms and ΔTE = 1.15 ms) B_0_ field map that was used in the offline image reconstruction pipeline.

On the Cima.X, similar data were collected with GRAPPA^18^ acceleration = 3 and either matched TE/TR as on the Achieva or with minimum TR ranging from 82 to 168 ms and minimum TE ranging from 62 to 95 ms, respectively. The latter data, with minimum TE/TR, were collected twice to assess the repeatability of an outlier.

Important to note is that the data acquisition for b‐values ≥ 4000 s/mm^2^ on the Philips Achieva system had to be collected in two packages (i.e., the whole volume was split in half and collected in two TRs, which was necessary to keep the effective TR the same as for lower b‐values).

Table 1 lists important acquisition parameters that vary between data acquisitions.

**TABLE 1:** Each number listed from left to right represents an acquisition parameter from data sets acquired with increasing b‐values of 500, 1000, 2000, 3000, 4000, 5000, and 10 000 s/mm^2^.

Parameters	3T Philips Achieva (No FM, w/ & w/o f0S)	3T Philips Achieva (FM)	3T Siemens Cima.X (Matched TR/TE)	3T Siemens Cima.X (Min TR/TE)
Slice TR [ms]	199	199	199	82,85,114,132,139,146,168
Packages	1,1,1,1,2,2,2	1,1,1,1,2,2,2	1	1
TE [ms]	76,87,103, 112,120,127,151	76,87,103,112,120,127,151	76,87,103,112,120,127,151	62,64,69,73 77,81,95
Coil	Philips 32ch	NeuroCam 16ch	Siemens 32ch	Siemens 32ch
Acceleration	SENSE 3	SENSE 3	GRAPPA 3	GRAPPA 3

*Note*: Cells with a single entry indicate that the same setting was used for all b‐values.

Abbreviations: ch, channel; FM, field monitoring; f0S, f0 stabilization; SENSE, sensitivity encoding; TE, echo time; TR, repetition time.

### 2.3 Data analysis and reconstruction

The NeuroCam data sets with concurrent field monitoring were reconstructed offline using an in‐house software pipeline that allows for higher‐order reconstruction of measured trajectories.^19^ After removing up to second‐order concomitant field contributions,^20^, ^21^ we used a full second order or full third spatial order of spherical harmonic (SpHa) field expansion to the trajectories as measured with the 16 NMR field probes.^22^ These SpHa fits were subsequently used for image reconstruction. We investigated the effects of two common spatial SpHa orders on the DIM, as it has been shown in some cases that a standard third‐order fit may not always provide the best image quality.^23^, ^24^ We observed unexpectedly large artifacts in the first diffusion direction of every second reconstructed slice due to an error in the field‐monitoring acquisition and reconstruction pipeline (likely related to number of TR packages for high b‐values). Rather than investigating the cause, we welcomed these image artifacts because they help demonstrate the sensitivity of DIM to a single image being corrupted in a large DWI data set. Three different analyses were carried out on these latter data: (i) excluding the first diffusion direction from all corrupted data sets, (ii) including the first but excluding the last diffusion direction (to keep the total amount of data comparable to the first analysis), and (iii) including all diffusion directions.

Moreover, we estimated the diffusivity of silicone oil within an 11 × 11 × 11 region of interest at the center of the phantom by computing the ratio of mean signal across diffusion directions to the corresponding signal in the b_0_ image and dividing this by the respective b‐value. For visualization, we also display the signal per diffusion direction for each b‐value together with its b_0_ image for two representative experiments in Figure S2.

Finally, we performed simulations with varying diffusivity values around that of the silicone oil phantom to quantify the effect of higher b‐values and diffusion on DIM (see Figure S3).

The data analyses were performed with custom‐made *MATLAB* scripts (version 2022b; The MathWorks, Inc., Natick, MA, USA).

All data sets were slice‐wise rigid body–registered (allowing only for translational shifts) to their respective first EPI volume (i.e., first diffusion direction).

### 2.4 Comparison and complementarity with a specific RAPID test

DIM does not directly reveal the cause of an existing instability. Rather, its benefit lies in identifying and quantifying instabilities; thus, it is a complementary test to other existing QA procedures in dMRI to help compare sites, scanners, data acquisition variants, and image‐reconstruction methods. To illustrate where reinforcing and complementary information can be gained, we also ran the linearity gradient checks from RAPID^13^ on the Achieva and Cima.X data sets (with minimal TR/TE) without concurrent field monitoring, which provides useful insight and visual comparison across different b‐values.

## 3 RESULTS

**FIGURE 2:**
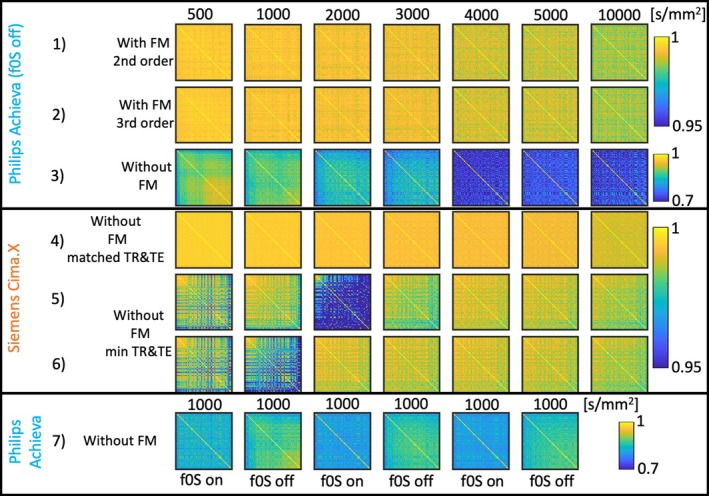
Correlation coefficient matrices for different data acquisitions. *Top two rows*: Correlation coefficient matrices calculated for diffusion directions 2 to 64 with increasing b‐values from left to right utilizing either second (*first row*) or third (*second row*) order spherical harmonics fits. *Third and fourth rows*: Same kind of visualization but for the Philips and Siemens data sets without field monitoring (FM) with matched TR/TE. *Fifth and sixth rows*: Siemens data set with minimal TR/TE. *Seventh row*: Same kind of visualization but for b‐value 1000 s/mm^2^ with f0S stabilization alternatingly turned off/on. Note that rows 1, 2, 4, 5, and 6 are windowed between [0.95, 1], whereas rows 3 and 7 are windowed between [0.7, 1].

Figure 2 shows the correlation coefficient matrices for all acquired HARDI data sets. There is a tendency to progressively reduce correlation coefficients as b‐value, TE, and TR increase. For any given setting, the correlation among DWIs can be recovered with the introduction of concurrent field monitoring or moving to a newer scanner with improved gradient performance.

**FIGURE 3:**
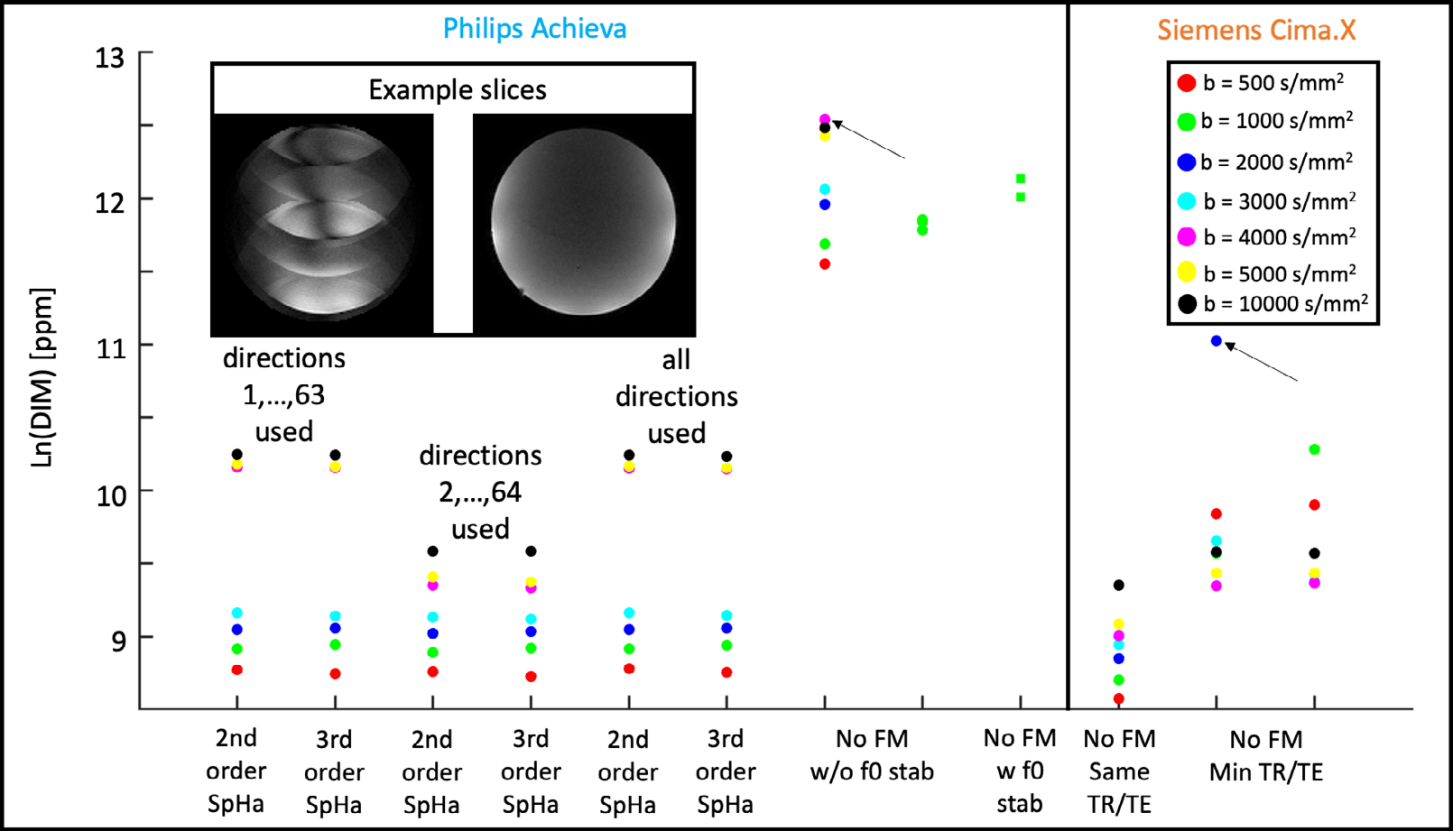
Diffusion instability measure (DIM) values for different data sets scaled logarithmically (base e). The first six columns on the left provide the DIM values for field‐monitored data sets with various b‐values and with second‐order and third‐order spherical harmonics reconstructions, calculated over diffusion directions 1–63 and 2–64 or all directions. The inset phantom slices show slice 30 of b = 4000 s/mm^2^ acquisition, where the first diffusion‐weighted image (DWI; *left*) has significant ghosting artifacts, but the 22nd DWI (*right*) is artifact‐free. The rightmost six columns provide the DIM without field monitoring (FM) for both the Philips (Columns 7–9) and the Siemens (Columns 10–12) scanner. At b = 1000 s/mm^2^, the Philips data were collected with/without center frequency stabilization. The Siemens data were collected with identical echo time (TE)/repetition time (TR) to that of the Philips scanner as well as with the minimum TE/TR that the Cima.X was capable of. The small black arrows only serve as a visual aid that point to the rather unexpected outliers in the data sets.

Figure 3 provides a scatter plot of the DIM results given in parts per million on logarithmic scale (base e) for all HARDI data sets. Unsurprisingly, in the Philips data set, the DIM values from the data with field‐monitoring correction are significantly lower, indicating improved image quality, than those without field monitoring. Excluding the artifact‐corrupted first DWI from the analysis also reduces DIM as expected. There is a negligible difference of DIM between the second‐order and third‐order spherical harmonic fits of the monitored trajectories, whereas there is a consistent gap between the lowest (≤ 3000 s/mm^2^) and the highest b‐values (≥ 4000 s/mm^2^) for all Philips data sets.

Another notable aspect is that without field monitoring, the data from the older Philips Achieva scanner model had significantly higher DIM values than that of the newer Siemens Cima.X scanner. This observation was true even for scans that were collected on the Cima.X with minimum TE/TR, which were both much shorter than that possible on the Achieva. However, when field monitoring was used on the Achieva scanner, data quality was significantly improved, as empirically evidenced by much lower DIM values.

**FIGURE 4:**
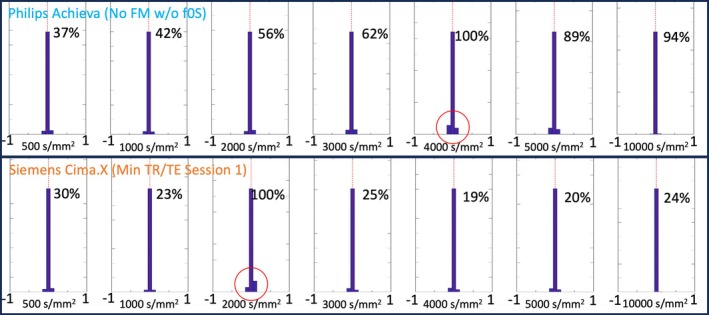
*Top row*: Gradient linearity residual histograms computed and displayed via RAPID^13^ for increasing b‐values (*left to right*) of the Philips No field monitoring (FM) and w/o f0S data sets. The percent numbers show the respective diffusion instability measure values scaled with respect to the outlier measurement (*encircled in red*). The red dotted line marks the 0 line of the histograms. *Bottom row*: Same kind of histograms but for the Siemens data sets with minimal repetition time (TR)/echo time (TE), first session.

Finally, Figure 4 shows the gradient linearity residual histograms (i.e., $\hat{S}[b]‐S[b]$, where $\hat{S}[b]$ are the fitted signal intensities) performed using the RAPID^13^ pipeline for the Philips data without field monitoring or center‐frequency stabilization (top row) as well as the Cima.X data with minimum TR/TE first‐session data sets (bottom row). Data from both scanners contained unexpected outliers (at 4000 or 2000 s/mm^2^ from the Achieva or Cima.X, respectively), which we used to scale the DIM values for all other data sets.

## 4 DISCUSSION

We proposed and tested an instability measure for dMRI data, whose calculation is analogous to the recently published TIM in fMRI,^14^ with the main differences being that the data input is a three‐dimensional set of DWIs instead of an fMRI time series and using a silicone oil instead of a gel phantom. DIM is obtained through the eigenratio of a correlation coefficient matrix, where each entry of the matrix is a correlation coefficient between the signal across all voxels of two DWIs with different diffusion‐encoding directions. DIM is easy to implement, can be used with different scanners, and does not require any special pulse sequences that are not provided by the manufacturer. Furthermore, it complements well the previously proposed quality assurance protocols, such as RAPID.^13^

Many QA protocols exist in MRI to test signal stability (in fMRI),^25^, ^26^ quantitative (e.g., T_1_ relaxation time)^27^ or geometric^28^ aspects of the acquired images, to name a few. However, fewer QA protocols exist for dMRI,^13^, ^29^, ^30^ with a tendency to use water (or other diffusing liquids^31^) for filling the phantom. There are several limitations with these protocols: (i) They inherently assume high quality of the underlying images without explicitly testing this, (ii) they infer only on the gradient linearity and calibration; and (iii) they can be used only with lower (max ˜3000 s/mm^2^) b‐values. In contrast, the silicone oil exhibits minimal diffusion (see Figure S2), with a mean value on the order of 10^−6^ across diffusion directions; thus, DWIs collected with b‐values of up to at least 10′000 s/mm^2^ can be used (see simulation results in Figure S3).

It should be noted that the temperature sensitivity of diffusion in silicone oil was not specifically tested, and possible temperature‐related effects were not considered. However, it is the authors' belief that at such low rate of diffusion and because MRI scanners are operating between tight specs of 19°C–22°C, temperature‐related effects are likely minimal.

High and stable signal intensity allows for inspecting and comparing the actual image quality of each DWI, regardless of diffusion direction or b‐value, and hence can reveal artifacts that can arise from other sources (e.g., RF instability or eddy current behavior), which are often exacerbated at higher b‐values and longer experiments.

Nevertheless, the importance of assessing gradient linearity and calibration is not contested here. Rather, DIM is proposed as a complementary measure, which, in combination with other QA protocols (e.g., the RAPID^13^), helps provide a more complete assessment of dMRI data quality than either of them alone.

DIM is specifically suited for uniform spherical phantoms, filled with silicone oil, where pixel intensities are expected to be identical across different diffusion directions. It is sensitive to sources of various system instabilities, such as varying ghost levels, parallel‐imaging reconstruction artifacts, RF transmit instability, or other sources that lead to loss in SNR. As such, DIM is a summary measure, which does not indicate the specific cause. Rather, it is a convenient summary metric for regular assessment of data quality. When it is found that DIM increases, the actual source of the issue can be identified with more specific tests.

As with temporal SNR in fMRI, there is no universally accepted threshold for DIM above which one can label the data as bad. DIM is best used to track scanner stability over time, compare scanners or sequences, or assess reproducibility across repeated experiments (see, for example, Figure 3 with b2000 on CimaX with minimal TR/TE).

Finally, we calculated the DIM after registration, which consistently resulted in a lower value than without registration (not shown) (i.e., registration helped removing spurious instabilities such as gradual drifts). Despite the change in numerical value, a thorough comparison can still be meaningful if the same processing steps are applied to each data set.

## 5 CONCLUSION

The DIM provides crucial complementary information on data quality that supports the outcome measures of other QA protocols in dMRI. The acquisition protocol and image processing pipeline of DIM are simple to implement for any scanner, without the need for pulse programming or special research contracts with the manufacturer, whereas the *MATLAB* code is available from the authors.

## CONFLICT OF INTEREST

Nothing to report.

## Supporting information


**Figure S1.** Simulated variability of DIM for different remaining voxel content, *N*_*v*_ ranging from 99 to 80% for four different representative datasets. The middle line shows the mean DIM across 100 random resamples at each percentage of remaining voxels, i.e. ${\bar{\mathrm{DIM}}}_{\text{remaining voxels}}=\frac{1}{100}\sum_{i=1}^{100}\mathrm{DIM}\left({\text{Data}}_{\text{remaining voxels}\left(i\right)}\right)$. The error bars indicate the limits of the standard deviation computed across the 100 resamples. Note that even for 80% remaining voxel content, the calculated DIM stays very stable.
**Figure S2.** Mean signal intensity for each diffusion direction is shown for representative experiments and two scanners: Philips No FM No f0S (top) and CimaX Matched TR/TE (bottom). The horizontal line represents the mean b0 signal (${\bar{S}}_{o}$), while the wavy line depicts the mean diffusion weighted signal (${\bar{S}}_{i}$) for each diffusion direction, *i* – both estimated within an 11x11x11 voxel ROI at the center of the phantom. Di5erent colors correspond to different b‐values. The color‐coded numbers next to “D=” are the corresponding estimated diffusivities, $D_{i}=-\frac{1}{b}\log\frac{{\bar{\bar{S}}}_{i}}{{\bar{S}}_{o}}$. Note that the y‐axes show arbitrary intensity units.
**Figure S3.** Simulation results using phantom diffusion data by taking the b_0_ signal from a representative dataset (here b500 Philips No FM No f0S) and multiplying it with exp (−bD) for different b‐values and diffusion constants, D, and finally adding gaussian noise to achieve different SNR levels. The colors red, yellow, green and blue correspond to the simulated diffusion constants 0, 1e‐7, 1e‐6, 1e‐5, respectively. The different marker shapes correspond to different SNR levels (although for small diffusion constants the markers overlap). As expected, DIM increases monotonically with decreasing SNR as well as increasing b‐value and diffusion constant but hardly varies for very low diffusion.
