# Supplementary material for: DIM: A diffusion instability measure for MRI quality assurance
Source: Magn Reson Med. 2025 Aug 10;94(6):2624–31. doi: 10.1002/mrm.70012 (PMC12501680; doi:10.1002/mrm.70012)
Supplement: Supplementary file 1 — Figure S1. Simulated variability of DIM for different remaining voxel content, N v ranging from 99 to 80% for four different representative datasets. The middle line shows the mean DIM across 100 random resamples at each percentage of remaining voxels, i.e. DIM‾remaining voxels=1100∑i=1100DIMDataremaining voxels(i). The error bars indicate the limits of the standard deviation computed across the 100 resamples. Note that even for 80% remaining voxel content, the calculated DIM stays very stable. Figure S2. Mean signal intensity for each diffusion direction is shown for representative experiments and two scanners: Philips No FM No f0S (top) and CimaX Matched TR/TE (bottom). The horizontal line represents the mean b0 signal (S‾o), while the wavy line depicts the mean diffusion weighted signal (S‾i) for each diffusion direction, i – both estimated within an 11x11x11 voxel ROI at the center of the phantom. Di5erent colors correspond to different b‐values. The color‐coded numbers next to “D=” are the corresponding estimated diffusivities, Di=−1blogS‾¯iS‾o. Note that the y‐axes show arbitrary intensity units. Figure S3. Simulation results using phantom diffusion data by taking the b0 signal from a representative dataset (here b500 Philips No FM No f0S) and multiplying it with exp (−bD) for different b‐values and diffusion constants, D, and finally adding gaussian noise to achieve different SNR levels. The colors red, yellow, green and blue correspond to the simulated diffusion constants 0, 1e‐7, 1e‐6, 1e‐5, respectively. The different marker shapes correspond to different SNR levels (although for small diffusion constants the markers overlap). As expected, DIM increases monotonically with decreasing SNR as well as increasing b‐value and diffusion constant but hardly varies for very low diffusion. [file MRM-94-2624-s001.pdf]

Supplementary Figures

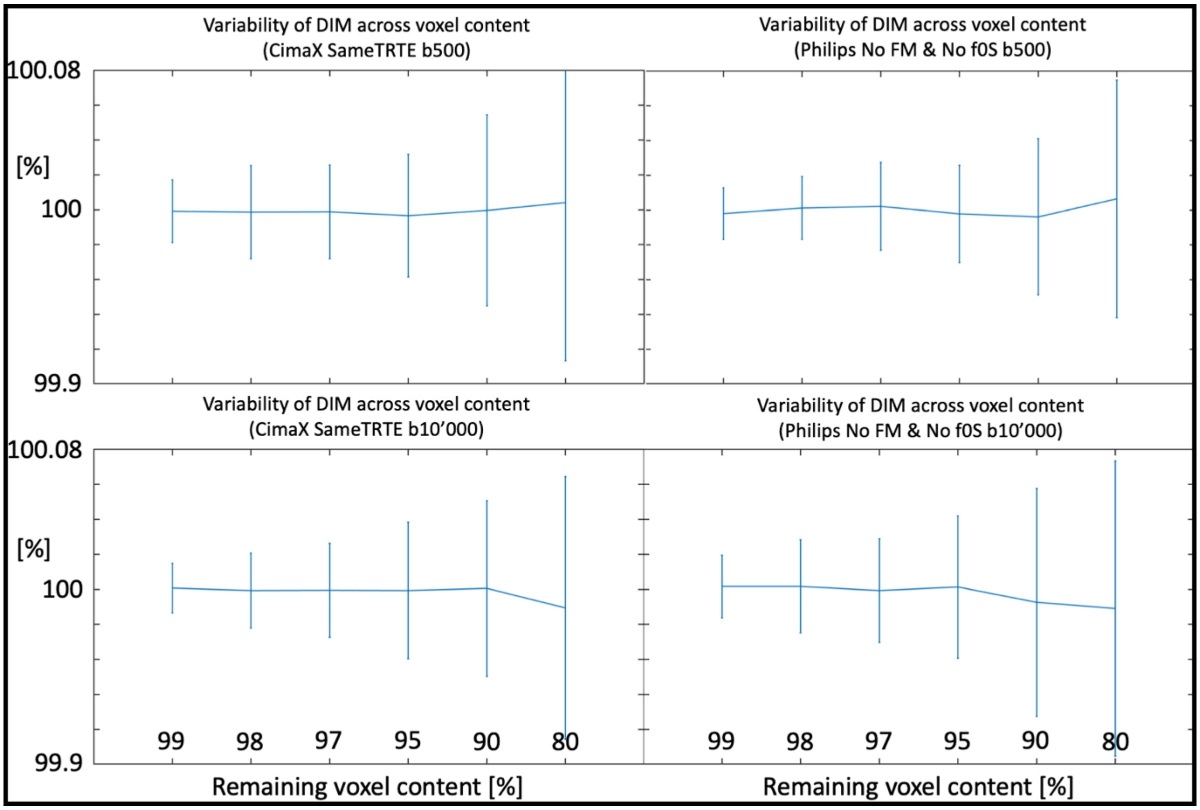

Figure S1. Simulated variability of DIM for different remaining voxel content, N_v ranging from 99 to 80% for four different representative datasets. The middle line shows the mean DIM across 100 random resamples at each percentage of remaining voxels, i.e. $\overline{DIM}_{remaining\ voxels} = \frac{1}{100}\sum_{i=1}^{100} DIM(\,Data_{remaining\ voxels(i)})$. The error bars indicate the limits of the standard deviation computed across the 100 resamples. Note that even for 80% remaining voxel content, the calculated DIM stays very stable.

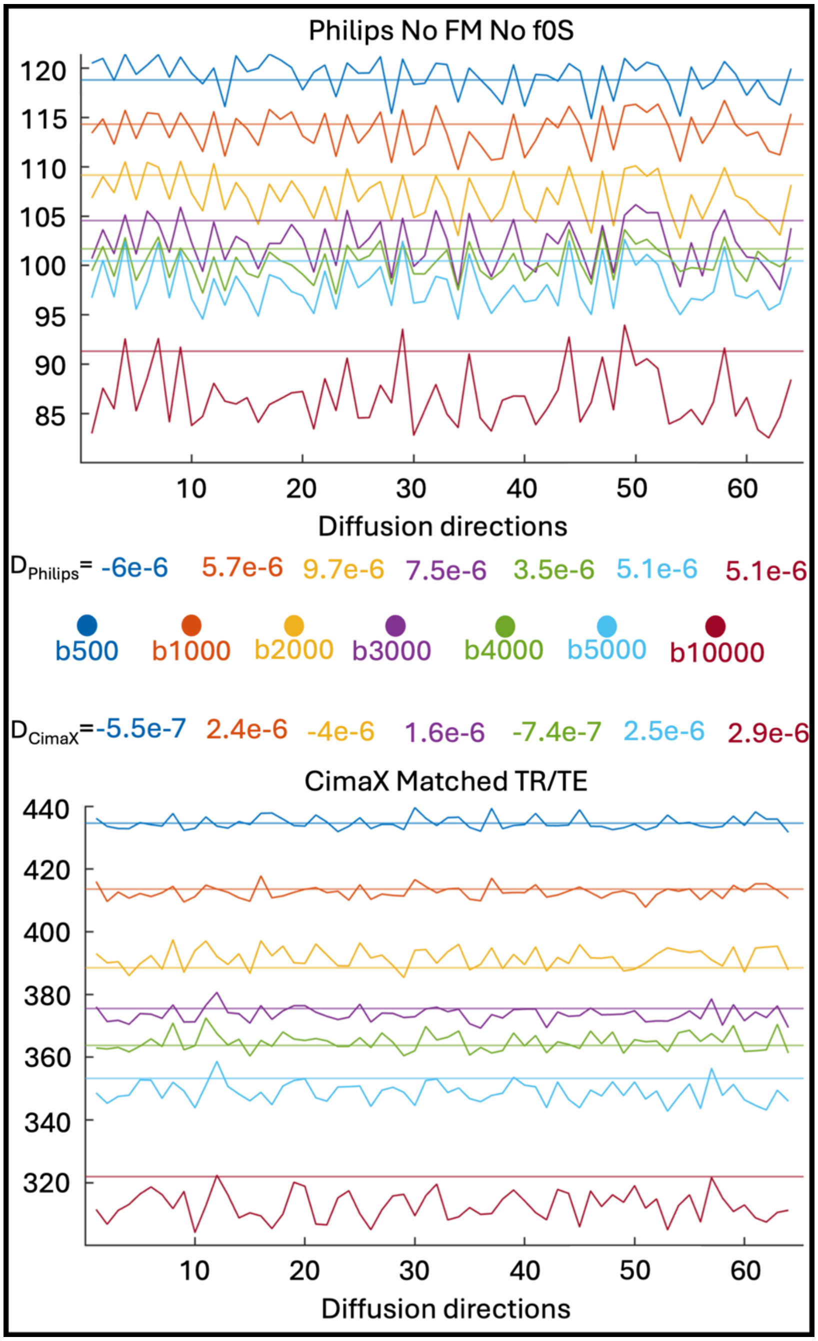

Figure S2. Mean signal intensity for each diffusion direction is shown for representative experiments and two scanners: Philips No FM No f0S (top) and CimaX Matched TR/TE (bottom). The horizontal line represents the mean b0 signal ($\bar{S}_o$), while the wavy line depicts the mean diffusion weighted signal ($\bar{S}_i$) for each diffusion direction, *i* – both estimated within an 11x11x11 voxel ROI at the center of the phantom. Different colors correspond to different b-values. The color-coded numbers next to "D=" are the corresponding estimated diffusivities, $D_i = -\frac{1}{b}\log\frac{\bar{S}_i}{\bar{S}_o}$. Note that the y-axes show arbitrary intensity units.

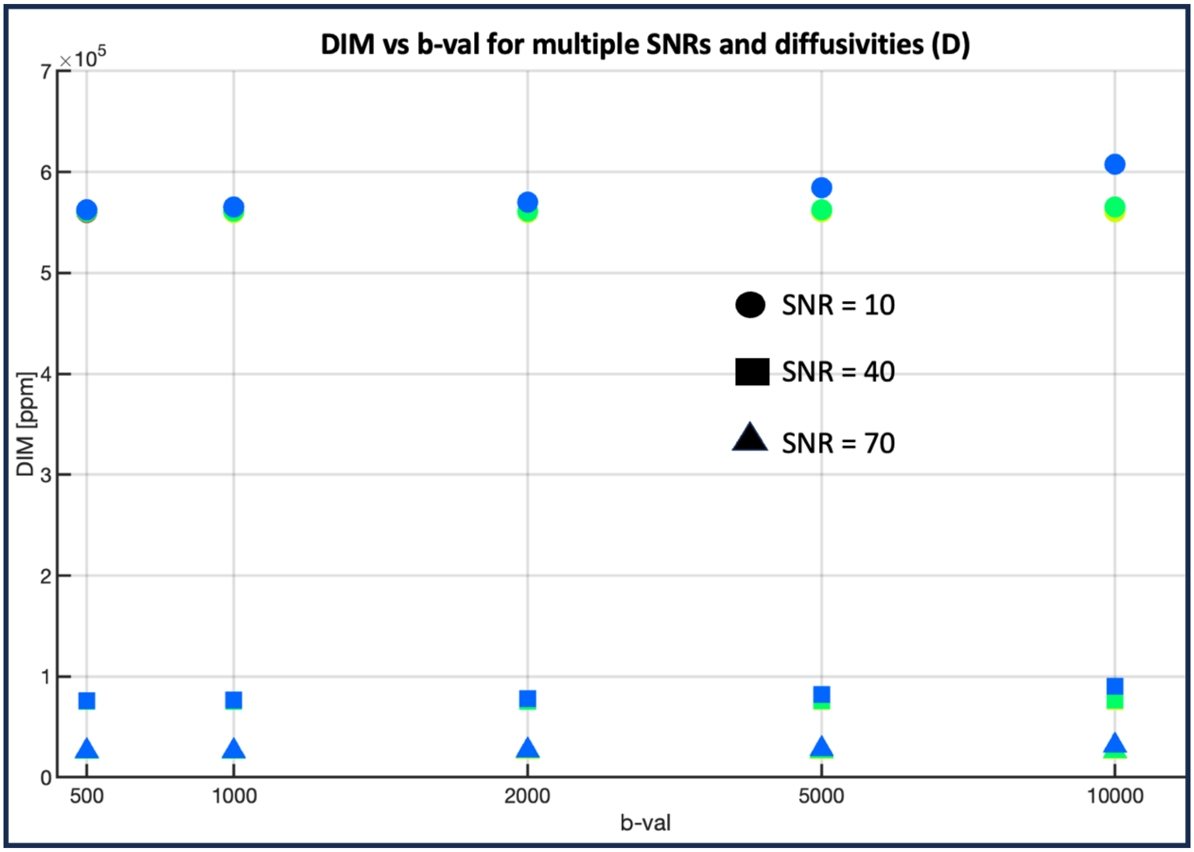

Figure S3. Simulation results using phantom diffusion data by taking the b_0 signal from a representative dataset (here b500 Philips No FM No f0S) and multiplying it with $\exp(-bD)$ for different b-values and diffusion constants, D, and finally adding gaussian noise to achieve different SNR levels. The colors red, yellow, green and blue correspond to the simulated diffusion constants 0, 1e-7, 1e-6, 1e-5, respectively. The different marker shapes correspond to different SNR levels (although for small diffusion constants the markers overlap). As expected, DIM increases monotonically with decreasing SNR as well as increasing b-value and diffusion constant but hardly varies for very low diffusion.
